# Comorbidity of Anxiety and Hypertension: Common Risk Factors and Potential Mechanisms

**DOI:** 10.1155/2023/9619388

**Published:** 2023-05-25

**Authors:** Tingting Qiu, Zhiming Jiang, Xuancai Chen, Yehua Dai, Hong Zhao

**Affiliations:** ^1^School of Nursing, Hengyang Medical School, University of South China, Hengyang, Hunan 421001, China; ^2^The Central Hospital of Changsha City, Hengyang Medical School, University of South China, Changsha, Hunan 410000, China; ^3^Department of Cardiology, The Fourth Hospital of Changsha, Changsha, Hunan 410006, China; ^4^Urinary Surgery, Affiliated Nanhua Hospital, University of South China, Hengyang 421002, China; ^5^Nursing College, University of Xiangnan, Chenzhou, Hunan 423000, China

## Abstract

Anxiety is more common in patients with hypertension, and these two conditions frequently coexist. Recently, more emphasis has been placed on determining etiology in patients with comorbid hypertension and anxiety. This review focuses on the common risk factors and potential mechanisms of comorbid hypertension and anxiety. Firstly, we analyze the common risk factors of comorbid hypertension and anxiety including age, smoking, alcohol abuse, obesity, lead, and traffic noise. The specific mechanisms underlying hypertension and anxiety were subsequently discussed, including interleukin (IL)-6 (IL-6), IL-17, reactive oxygen species (ROS), and gut dysbiosis. Increased IL-6, IL-17, and ROS accelerate the development of hypertension and anxiety. Gut dysbiosis leads to hypertension and anxiety by reducing short-chain fatty acids, vitamin D, and 5-hydroxytryptamine (5-HT), and increasing trimethylamine N-oxide (TAMO) and MYC. These shared risk factors and potential mechanisms may provide an effective strategy for treating and preventing hypertension and comorbid anxiety.

## 1. Introduction

Hypertension is the leading preventable risk factor for cardiovascular disease and all-cause mortality globally [1]. The prevalence of hypertension projected to exceed 1.5 billion by 2025 [2]. Hypertension affects approximately 1 billion adults and associated with 9 million global fatalities annually [3]. Besides hypertension, mental disorders are serious public health concern [4]. According to a meta-analysis, the incidence of comorbid hypertension and anxiety is approximately 38% [5].

Anxiety disorders are the most common category of mental disorders. The global prevalence of anxiety disorders is approximately 7.3%, accounting for 3.3% of the global disease [6]. The World Health Organization ranked anxiety disorders as the ninth leading cause of disability due to their high prevalence, chronicity, and comorbidity [7]. Numerous studies have reported a positive association between hypertension and anxiety. Comorbidities, such as hypertension and anxiety are associated with lower treatment compliance, lower levels of daily functioning, lower health-related quality of life, and healthcare-related costs [8]. Furthermore, patients with hypertension are at a greater risk of cardiovascular disease-related mortality [9, 10].

Here, we reviewed the relationship between hypertension and anxiety. Additionally, we discuss some of the most prevalent risk factors and potential mechanisms of comorbid hypertension and anxiety.

## 2. Positive Association between Hypertension and Anxiety

Recent research shows that the severity of disability is higher among patients with comorbid anxiety disorders and hypertension. Hence, the relationship between anxiety and hypertension is worth investigating in depth to develop comprehensive and integrated interventions to reduce disability [11].

The relationship between hypertension and anxiety is mutually reinforced. Longitudinal data and theoretical literature indicate that anxiety is an etiology for hypertension [12]. Anxiety is an independent risk factor of hypertension and promotes the development and progression of the condition. Patients with hypertension are prone to anxiety due to the disease or inefficacious treatment [13]. Anxiety disorders were more prevalent in patients with hypertension (37.9%) than in the general population (12.4%) [14]. Additionally, anxiety is an independent risk factor for hypertension [15] due to its influence on unhealthy behaviors such as smoking, depression, a sedentary lifestyle, and being overweight [16]. Anxiety was more prevalent among older hypertensive patients with a medical history of stroke and depression [17]. Like essential hypertension, anxiety also consistently increases the risk of hypertensive disorders during pregnancy and eclampsia [18]. Accordingly, anxiety increases the probability of developing hypertension.

Until recently, research has suggested that older adults with comorbid hypertension and anxiety have an increased probability of medication noncompliance, resulting in decreased treatment efficacy [13]. Based on the preceding data, anxiety may be a barrier to medication adherence, exacerbating hypertension, and cardiovascular complications. Anxiety significantly affects the functional, cognitive, and affective dimensions of quality of life in patients with hypertension. Furthermore, individuals with hypertension incur an additional excess economic burden from anxiety [8]. Overall, anxiety is directly related to the life of patients with hypertension. Antianxiety treatment is effective in lowering blood pressure in hypertension patients [12]. Therefore, early detection and treatment of comorbid hypertension and anxiety are critical.

## 3. Risk Factors for Comorbid Hypertension and Anxiety

The co-occurrence of hypertension and anxiety suggests the existence of mutual risk factors. Individual risk factors, lifestyle choices, and environmental risk factors (Table 1) are the primary risk factors associated with comorbid hypertension, according to a previous study. In this study, we analyzed the effects of these risk factors on the incidence of comorbid hypertension and anxiety.

### 3.1. Age

Age is a specific risk factor. Age was the most significant individual risk factor for comorbid hypertension related anxiety. Significant evidence indicates that the risk of hypertension increases with age [39]. The prevalence of hypertension is 26% in people aged 20–44, compared to 78% among those >65 years of age [19]. Vascular endothelial cells (ECs) are widely known to play an important role in vascular homeostasis and tone modulation. With increasing age, ECs undergo vascular senescence, which exacerbates apoptosis and inflammation, resulting in increased arterial stiffness and loss of endothelial cell-induced vasodilation, thereby increasing the risk of hypertension [20]. Age enhances the severity of anxiety similar to hypertension [21]. The fundamental cause could be that the number of 5-hydroxytryptamine (5-HT) specific receptors in the brain may be significantly reduced, increasing the probability of anxiety development [22]. 5-HT also promotes vasodilation by activating the 5-HT receptors in the endothelium to promote NO production. This implies that reducing 5-HT receptors may contribute to endothelial dysfunction [40]. Moreover, older adults with a higher prevalence of hypertension are more vulnerable to comorbid anxiety disorders [41]. Therefore, age is a primary risk factor for comorbid hypertension and anxiety.

### 3.2. Sex

Sex also plays a role in the development of hypertension. Specifically, reproductive hormones, such as estrogen and testosterone, likely contribute to sex differences in blood pressure (BP) and anxiety [42]. Premenopausal women generally have beneficial metabolic, cardiovascular, and sympathetic profiles under estrogen protective conditions [43]. However, the loss of estrogen due to menopause in women during midlife and older age could increase their susceptibility to “central obesity” and hypertension [23]. Similarly, women were twice as likely as men to experience anxiety [24]. Several lines of evidence indicate that “withdrawal” or decrease in estrogen during natural hormonal shifts (within the menstrual cycle, postpartum, and during the menopause transition) increases anxiety risk [25]. Therefore, understanding the sex differences in cardiometabolic and mental risk factors is important for selecting preventive and/or therapeutic strategies in both men and women.

### 3.3. Lifestyle

Undesirable lifestyle factors, such as smoking, alcohol abuse, and obesity, are crucial in comorbid hypertension and anxiety. First, chronic exposure to tobacco smoke alters the autonomic nervous system's regulation of BP, resulting in hypertension due to an unrestrained increase in sympathetic activation [26]. Furthermore, cigarette smoke contains chemicals that activate oxidative stress. Hypertension is caused by vascular inflammation and aging due to increased superoxide generation [29]. Strikingly, there was a strong link between smoking >20 cigarettes per day and an elevated risk of anxiety [27]. Interestingly, smoking exacerbates anxiety in many ways, including nicotine withdrawal symptoms, perceived and real health impairments, and physical illness [28]. Second, alcohol elevates BP in a dose-dependent manner. Heavy alcohol consumption increases the risk of hypertension by altering the heart or vascular smooth muscle and stimulating the sympathetic nervous system or the renin-angiotensin-aldosterone (RAS) system [30]. Alcohol abuse can exacerbate anxiety. Chronic alcohol abuse alters brain physiology, resulting in psychological sequelae. Furthermore, a paucity of coping responses and a sense of helplessness, inadequacy, and anxiety can be caused by alcohol intoxication during stressful situations [31]. Furthermore, alcohol abuse increases the engulfment capacity of microglia, causing aberrant synaptic pruning, synapse loss, and anxiety-like behaviors [32]. Third, obesity is a significant predictor of various chronic disorders. Numerous epidemiological studies have demonstrated that being overweight or obese causes 65%–75% of primary hypertension cases. Obesity-related hypertension is initiated by impaired kidney function and increased tubular salt reabsorption [33]. Obesity is associated with various neurodegenerative disorders, particularly anxiety [34]. The primary reason is obesogenic lifestyle factors such as dense caloric diets and inactivity. Mechanically, senescent glial cells exhibit excessive fat deposition, and obesity causes glial cell senescence, which generates anxiety [44]. Collectively, lifestyle risk factors are indispensable in the development of comorbid hypertension and anxiety.

### 3.4. Environment

Environmental risk factors are associated with the exposure to Pb and traffic noise. Lead, one of the most common environmental stressors, can cause irreversible damage to the central nervous system, especially during development. Extended and desultory exposure to lead causes neuroinflammation, characterized by astroglial and microglial gliosis. Both anxiety and hypertension are caused by neuroinflammatory processes that develop over time [35]. In addition, hypertension can be caused by baroreceptor and chemoreceptor reflex dysfunction due to intermittent or persistent lead exposure [36]. A recent study suggested that lead exposure generates oxidative stress and induces gut dysbiosis, which can lead to hypertension and anxiety [45]. Consequently, people exposed to lead are at high risk for comorbid hypertension and anxiety, especially those with occupational exposure.

Traffic noise (from roads, aircraft, and railways) is a potential risk factor for cardiovascular and mental illnesses [46]. Traffic noise disrupts and shortens sleep, elevates stress hormone levels, and causes severe oxidative stress in blood vessels and the brain. These compounds facilitate inflammation, endothelial dysfunction, and hypertension [37]. Increased exposure to traffic noise is considered a significant risk factor for anxiety. It is generally recognized that traffic noise is regarded as a formidable neurological stressor [38]. Overall, environmental risk factors negatively impact the cardiovascular and neurological systems during the early stages of development. It is imperative to implement public policy measures to mitigate the negative consequences of these environmental risk factors.

Age, sex, smoking, alcohol abuse, obesity, lead exposure, and traffic noise contribute to the development of comorbid hypertension and anxiety. Preventive measures such as risk factor screening and early management can enhance patient well-being. More importantly, the specific mechanisms involved in comorbid hypertension and anxiety require further exploration.

## 4. Potential Mechanisms of Comorbid Hypertension and Anxiety

### 4.1. IL-6

Interleukin-6 (IL-6) stimulates chronic inflammation and regulates the expression of C-reactive protein and cardiovascular disease risk biomarker [47]. IL-6 is related to angiotensin II (Ang-II)-mediated hypertension [48]. IL-6 is enhanced in response to exogenous Ang-II infusion, which increases the activity of the RAS system and ultimately causes hypertension to occur and develop [49]. Furthermore, IL-6 stimulates IL-17 production by inducing the polarization of CD^4+^ T cells, resulting in hypertension [50, 51]. IL-6 may trigger obesity by inducing hepatic gluconeogenesis and inhibiting lipid metabolism, thereby facilitating hypertension [52].

Similarly, individuals with anxiety disorders have elevated circulating IL-6 levels [53]. IL-6 levels are elevated in blood during repeated social defeat [54]. Increased IL-6 levels activate the bone marrow-derived peripheral monocytes and promote their recruitment to neurovascular endothelial cells, thereby releasing IL-1*β* and triggering anxiety [55, 56]. Because of reduced amygdala volumes, maternal IL-6 levels can predict offspring anxiety [57]. Therefore, IL-6 levels are closely associated with anxiety. Here, the functions of IL-6 in hypertension and anxiety were unclear. Despite the abundance of available studies, more experiments are required to understand the pathophysiological effects of IL-6.

### 4.2. IL-17

IL-17 induces the phosphorylation of endothelial nitric oxide (NO) synthase (eNOS) on threonine 495 in a Rho kinase-dependent manner, resulting in decreased NO generation and the development of hypertension [58]. Additionally, IL-17 reduces lumen diameter and increases wall thickness, which induces inward hypertrophy and arterial stiffness and results in hypertension [59].

IL-17 promotes anxiety development through neuronal IL-17A receptor (IL-17Ra) signaling in the medial prefrontal cortex [60]. Additionally, the astrocyte markers glial fibrillary acidic protein (GFAP) and brain-derived neurotrophic factor (BDNF) expression were also reduced by IL-17 [61]. Reductions in both GFAP and BDNF levels are vital mediators for the advancement of anxiety [62, 63]. Consequently, we hypothesize that IL-17 is involved in the corporate etiology of comorbid anxiety and hypertension.

### 4.3. Reactive Oxygen Species (ROS)

Nicotinamide adenine dinucleotide phosphate oxidase (NOX) and mitochondria are the major enzymatic sources of ROS in the cardiovascular system [64]. The production of ROS by NOX promotes to mitochondrial ROS production by causing mitochondrial DNA damage and oxidation of components of the membrane permeability transition pores [65]. Moreover, excessive ROS generation damages biological components, such as nucleic acids, proteins, and lipids, leading to cell damage and death. Several human diseases, including cancer, Alzheimer's disease, hypertension, and anxiety, are believed to be caused by these mechanisms [66]. Moreover, abundant ROS and inflammatory factors, including IL-6 and tumor necrosis factor-*α* (TNF-*α*), contribute to comorbid hypertension and anxiety in rats [67]. Antihypertensive drugs (candesartan and azelnidipine) significantly reduce ROS and IL-6 levels [68, 69]. Additionally, candesartan improves BP and hippocampal neurogenesis, and is an efficacious treatment for cardiovascular disease and mental illness [69].

#### 4.3.1. ROS Accelerates the Development of Hypertension

ROS levels are also closely associated with endothelial dysfunction. ROS generation inhibits dihydrofolate reductase (DHFR) activity. DHFR deficiency led to mitochondrial ROS production. Excessive mitochondrial ROS production reduces NO bioavailability which leads to endothelial dysfunction [70]. Similarly, the reduction in DHFR decreases tetrahydrobiopterin (BH4), an essential cofactor for eNOS. This leads to eNOS uncoupling, which produces superoxide rather than NO [71]. Moreover, ROS increases the expression of poly (ADP-ribose) polymerase 1 (PARP1) expression, a key activator of transient receptor potential melastatin 2 (TRPM2). Importantly, activated TRPM2 contributes to vascular Ca^2+^ influx through Na^+^/Ca^2+^ exchange (NCX) [72]. Hypertension is caused by excessive free intracellular Ca^2+^ concentration, which leads to endothelial dysfunction, vascular hyperreactivity, and structural remodeling [73]. Furthermore, ROS increases the response of thioredoxin-interacting protein (TXNIP), which in turn activates the NLR-family pyrin domain-containing protein 3 (NLRP3). By provoking endothelial inflammation, NLRP3 activation worsens endothelial dysfunction [74].

ROS-mediated hypertension is complicated by the endoplasmic reticulum (ER) involvement. ROS generation activates ER stress signaling pathways involving transcription factor 6 (ATF6), inositol-requiring protein 1 (IRE-1), and PRKR-like ER kinase [75]. Moreover, ER stress has been shown to increase TGF-1 activity, decrease phosphorylation of endothelial NO synthases, and worsen endothelium-dependent relaxation contributing to hypertension [76].

**Figure 1:**
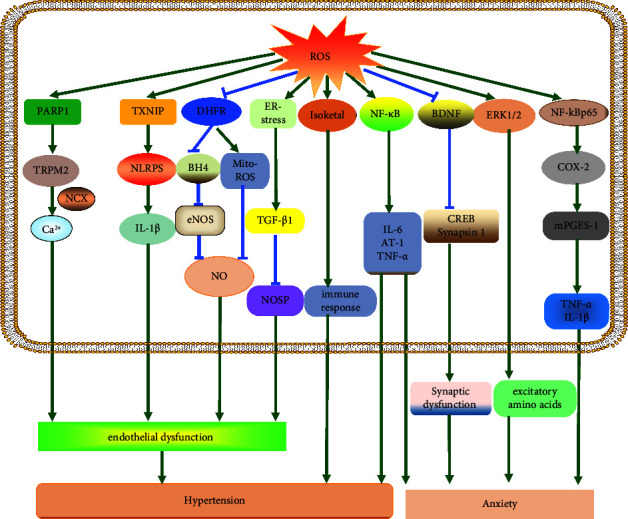
ROS promotes the pathogenesis of hypertension and anxiety.

Moreover, ROS is closely associated with hypertension as they promote the formation of isoketals. Hypertensive individuals exhibit significant isoketone expression [77].ROS induces the formation of isoketals, accumulating in dendritic cells (DCs). Accumulation of isoketals promote DC activation of T cells, which release IL-17 and IFN-*γ* that cause aortic stiffening, ultimately leading to overt hypertension [78]. Additionally, increased ROS levels are caused by many maternal conditions, such as obesity and smoking. Superfluous ROS then causes gut dysbiosis and RAS dysfunction in offspring, contributing to the generational programming of hypertension in adult offspring [79]. Therefore, ROS facilitates the incidence and development of hypertension (Figure 1).

ROS leads to vascular Ca^2+^ influx by activating the PARP1-TRPM2 signaling pathway, inducing endothelial dysfunction, subsequently leading to hypertension. ROS promotes endothelial dysfunction by activating TXNIP-NLRP3-IL-1*β* signaling pathway, causing hypertension. ROS reduces the production of NO by inhibiting DHFR-BH4-eNOS signaling pathway, exacerbating endothelial dysfunction, thus leading to hypertension. ROS causes mitochondria ROS generation by inhibiting DHFR expression, reducing the production of NO to exacerbate endothelial dysfunction, leading to hypertension. ROS activates the ER stress enhancing TGF-*β*1 activity, reducing endothelial NO synthase phosphorylation to promote hypertension. ROS promotes immune response by inducing isoketal production, resulting in hypertension. ROS promotes IL-6, TNF-*α*, and AT-1expression by activating NF-*κ*B leading to hypertension and anxiety. ROS induces synaptic dysfunction by suppressing the BDNF-synapsin I/CREB pathway to aggravate anxiety. ROS enhances the excitatory amino acids release via inducing ERK1/2 resulting in anxiety. ROS induces the production of TNF-*α* and IL-1*β* by activating the NFkBp65-COX2-mPGES-1 signaling pathway, thus accelerating anxiety. PARP1: poly [ADP-ribose] polymerase 1; TRPM2: transient receptor potential melastatin 2; TXNIP: thioredoxin-interacting protein; NLRP3: NLR-family pyrin domain-containing protein 3; DHFR: dihydrofolate reductase; BH4: tetrahydrobiopterin; eNOS: endothelial nitric oxide synthase; AT-1: angiotensin 1; BDNF: brain-derived neurotrophic factor; CREB: incyclic AMP responsive element binding protein; ERK1/2: extracellular signal-regulated kinase1/2; NFkBp65: nuclear factor-kappa B p65; COX-2: cyclooxygenase-2; mPGES-1: microsomal prostaglandin synthase-1.

#### 4.3.2. ROS Promotes the Occurrence of Anxiety

Synaptic dysfunction refers to imbalanced synaptic plasticity due to disturbances in pre- and postsynaptic sites. Synaptic dysfunction mediates anxiety [80]. Recent research has identified that ROS accumulation may contribute to imbalanced synaptic function by reducing the expression of BDNF and downstream effector synapsin I molecules and intracyclic/incyclic AMP responsive element binding protein (CREB) [81]. Moreover, ROS induces hyperphosphorylation of extracellular signal-regulated kinase 1/2(ERK1/2). ERK1/2 hyperphosphorylation increases the release of excitatory amino acids resulting in anxiety [82]. Furthermore, ROS elevates nuclear factor-kappa B p65 (NF-kB p65), which activates the inflammatory mediators cyclooxygenase-2 (COX-2) and microsomal prostaglandin synthase-1 (mPGES-1) and causes an inflammatory response [83]. TNF-*α* and IL-1 production is aided by mPGES-1, which functions in conjunction with COX-2 to trigger anxiety [84].

Glyoxalase (GLO)-1 and glutathione reductase (GSR)-1 are specific antioxidant enzymes. ROS decreases the expression of (GLO)-1 and (GSR)-1 [82], and further enhances the level of anxiety by increasing inflammation and synaptic plasticity [85]. Amazingly, ROS upregulates the expression of IL-6 and TNF-*α* and angiotensin (AT)-1 receptors by activating NF-*κ*B generation. This process is involved in the development of hypertension and anxiety [67] (Figure 1). Given the detrimental roles of ROS in hypertension and anxiety, targeting ROS is a potential therapeutic strategy.

### 4.4. Gut Microbiota

The gut microbiotas play a vital role in promoting health. Gut microbiotas are microorganisms that colonize the digestive tract. Their functions include the digestion of food, elimination of toxins, regulation of immunity, regulation of metabolism, and induction of neurogenesis [86–88]. Gut dysbiosis plays a decisive role in disease susceptibility. Microbial communities can be disrupted by nutrition, long-term antibiotic use, stress, and gastrointestinal disorders. This process is referred to as gut dysbiosis. Individuals with gut dysbiosis, are characterized by the loss of microbial diversity and beneficial microorganisms and an increase in potentially harmful microbes [89]. Recent studies confirmed the involvement of gut dysbiosis in the onset and progression of various diseases. Gut dysbiosis is particularly common in individuals with colorectal cancer [90], neurodegenerative disorders [91], kidney disease [92], obesity, type 2 diabetes, or arterial stiffness [93].

**Figure 2:**
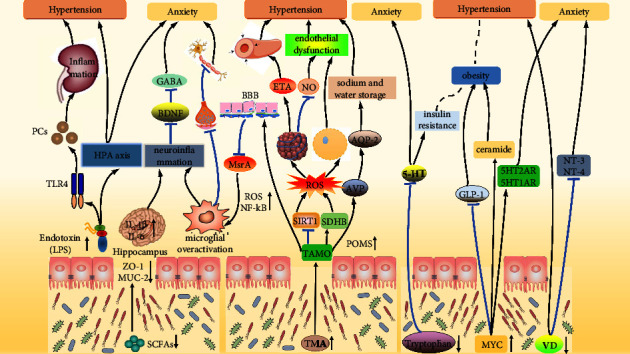
Gut dysbiosis accelerates the occurrence of hypertension and anxiety.

Notably, the gut microbiotas of both prehypertensive and hypertensive populations showed decreased microbial richness and diversity than healthy populations [94]. Furthermore, considerable evidence supports the involvement of gut dysbiosis in exacerbating hypertension [95, 96] because gut dysbiosis affects an individual's immune system and metabolism [97]. Additionally, gut dysbiosis contributes to anxiety by microbiota–gut–brain axis, which activates the vagus nerve and increases microbial metabolite production [87]. Changes in microbial metabolites, such as short-chain fatty acids (SCFs), trimethylamine N-oxide (TAMO), vitamin D, and 5-hydroxytryptamine (5-HT), play an indispensable role in the onset of hypertension and anxiety (Figure 2).

Gut dysbiosis leads to a reduction of SCFAs, disrupting the intestinal barrier by decreasing ZO-1 and MUC-2. The deficiency of SCFAs promotes the production of PCs by stimulating the LPS-TLR4 pathway, inducing renal inflammation leading to hypertension. The deficiency of SCFAs causes the overactivation of HPA axis by increasing the level of LPS, resulting in hypertension and anxiety. The deficiency of SCFAs exaggerates neuroinflammation by increasing IL-1*β* and IL-6 expression in the hippocampus, thereby promoting anxiety through inhibiting BDNF-GABA signal pathway. The deficiency of SCFAs reduces synapses via expediting microglial overactivation, inhibiting nerve conduction, and causing anxiety. Gut dysbiosis promotes the release of TMA into the bloodstream, further oxidized to TMAO. TMAO penetrates BBB, subsequently inhibiting MsrA expression promoting microglial overactivation, causing anxiety. TMAO promotes SDHB expression and inhibits SIRT1 expression, leading to ROS generation, which stimulates Ox-LDL secretion, reducing the production of NO, inducing endothelial dysfunction leading to hypertension. ROS stimulates Ox-LDL secretion, thus promoting vasoconstriction by increasing ETA, leading to hypertension. ROS promotes the pyroptosis of endothelial cells, causing endothelial dysfunction leading to hypertension. TMAO induces hypertension through the “TMAO-AVP-AQP-2 axis.” Gut dysbiosis suppresses 5-HT secretion by inhibiting tryptophan secretion, causing anxiety. The suppression of 5-HT exacerbates insulin resistance, contributing to obesity leading to hypertension. Gut dysbiosis increases intestinal MYC expression, facilitating obesity by decreasing GLP-1 secretion and stimulating ceramide synthesis, leading to hypertension. MYC accelerates anxiety by promoting the expression of 5HT2AR/5HT1AR. Gut dysbiosis contributes to hypertension by limiting VD production. The lack of VD results in anxiety by restraining the expression of NT-3/4. ZO-1: zonula occludens-1; MUC-2: mucoprotein-2; TLR4: toll-like receptor 4; MsrA: antioxidant enzyme methionine sulfoxide reductase A; SDHB: succinate dehydrogenase B; SIRT1: sirtuin 1; ETA: endothelial receptor A; AVP: pituitrin; AQP-2: aquaporin-2; GLP-1: glucagon-like peptide 1; ChREBP: carbohydrate-responsive element-binding protein; GLUT2: solute carrier family 2, member 2; SGLT1: solute carrier family 5, member 1; NT-3: neurotrophin-3; NT-4: neurotrophin-4.

#### 4.4.1. Short-Chain Fatty Acids (SCFAs)

Short-chain fatty acids (SCFAs) comprise butyrate, propionate, and acetate, produced by numerous bacterial species during dietary fermentation in the colon [98]. SCFAs increase energy expenditure and improve glucose metabolism and insulin secretion, reducing the risk of obesity, diabetes, metabolic liver disease, and cardiometabolic diseases [99]. Gut dysbiosis reduces SCFA production by decreasing beneficial bacteria such as Firmicutes and Bacteroidetes [100]. The lack of SCFs has been implicated in comorbid hypertension and anxiety [101]. Insufficient SCFAs promote hypertension by increasing intestinal permeability and the levels of proinflammatory cytokines and lipopolysaccharides (LPS). First, reducing SCFAs significantly decreases colonic zonula occludens-1 (ZO-1) and mucoprotein-2 (MUC-2). Deficiencies in ZO-1 and MUC-2 indicate a disrupted intestinal barrier and increased gut permeability, respectively [102]. Second, reducing SCFAs leads to an increase in proinflammatory cytokines, including TGF-*β*1, TNF-*α*, IL-1*β*, and IL-6, which in turn causes a reduction in glomerular filtration rate and consequently results in hypertension [103, 104]. Furthermore, insufficient SCFAs increase glucagon-like peptide 1 (GLP1) and gut hormone peptide YY (PYY), promoting Na^+^ absorption and volume overload in hypertension [105]. Third, SCFA deficiency increased the plasma levels of LPS. LPS stimulates toll-like receptor 4 (TLR4), which induces the production of proinflammatory cytokines involved in the pathogenesis of hypertension and anxiety [106, 107]. Likewise, hypertension and anxiety can develop due to of LPS activation of the hypothalamic–pituitary–adrenal axis (HPA axis) [108], which, in turn, promotes hypothalamic overactivity [109, 110].

Similarly, the absence of SCFAs causes anxiety by reducing *γ*-aminobutyric acid (GABA) and promoting microglial overactivation. The absence of SCFAs potentiates neuroinflammatory responses by enhancing the expression of proinflammatory cytokines (IL-1*β* and IL-6) in the hippocampus. Significantly, exaggerated neuroinflammation arrests t BDNF expression [111]. Moreover, reduced GABAergic transmission is the way that BNDF deprivation promotes anxiety [112]. In addition, the absence of SCFAs expedites microglial overactivation, which reduces the number of synapses between neurons in the prefrontal cortex, thereby inhibiting nerve conduction and causing anxiety [32]. The supplementation of SCFAs lowers BP [113] and anxiety levels [114]. Hence, SCFAs are potential therapeutic targets for comorbid hypertension and anxiety.

#### 4.4.2. Trimethylamine N-Oxide (TAMO)

Gut dysbiosis causes occasional hypertension because of excessive TAMO levels. Gut dysbiosis is characterized by increased harmful microbes, particularly those involved in choline degradation. Notably, choline degradation is widely observed in patients with hypertension, including *Prevotella*, *Klebsiella*, *Clostridium*, and *Streptococcus* [115]. Choline degradation produces trimethylamine (TMA) by metabolizing dietary choline, phosphatidylcholine, and l-carnitine. As mentioned previously, increased gut permeability promotes TMA release into the bloodstream. Subsequently, TMA is further oxidized to TMAO by flavin-dependent monooxygenases 1 (FMO-1) and FMO-3 [116]. Importantly, elevated plasma TMAO levels prolonged the hypertensive effects of Ang-II [117]. Specifically, elevated circulating TMAO induces hypertension through the “TMAO-AVP-AQP-2 axis.” Specifically, the release of pituitrin (AVP) was stimulated by an increase in plasma osmotic pressure (POM) caused by elevated TMAO. AVP increases the production of aquaporin-2 (AQP-2) in the apical membrane of the renal collecting duct's main cell, and sodium and water storage [118]. Moreover, TMAO induces oxidative stress by repressing sirtuin 1 (SIRT1) expression, subsequently impairing endothelial-dependent NO production [119]. Furthermore, TMAO impairs mitochondrial structure and increases ROS levels generation by upregulating succinate dehydrogenase B (SDHB) expression. Consequently, an increase in ROS promotes endothelial dysfunction by triggering pyroptosis of ECs [120]. Additionally, excessive ROS levels enhance the production of oxidized low density lipoproteins (Ox-LDL). Enhanced Ox-LDL increases endothelin-1 concentration, which activates endothelial receptor A (ETA), causing vasoconstriction and hypertension [121]. However, TMAO inhibition ameliorated hypertension by reducing neuroinflammation and oxidative stress [122].

TMAO is also a critical contributor to anxiety. In the hippocampus, TMAO inhibits the antioxidant enzyme methionine sulfoxide reductase A (MsrA) by penetrating the blood-brain barrier. The absence of MsrA in microglia promotes microglial overactivation by enhancing ROS production and NF-kB activity [123, 124]. Moreover, the probiotic application reduces TAMO levels, further alleviating anxiety in anxious patients [125]. This evidence suggests that TMAO plays a crucial role in the progression of hypertension and anxiety.

#### 4.4.3. Vitamin D

Hypertension and anxiety are more common in individuals with vitamin D deficiency [126]. Gut dysbiosis is closely associated with hypertension and anxiety by inhibiting vitamin D production. Gut dysbiosis decreases vitamin D levels, increasing the risk of hypertension [127]. The mechanisms underlying vitamin D deficiency-related hypertension include increased renin expression, hypocalcemia, and hyperparathyroidism [128]. Additionally, vitamin D disrupts the integrity of neurons by downregulating neurotrophic factors (neurotrophin-3 and neurotrophin-4) in the hippocampus and neocortex, causing anxiety [129]. Thus, vitamin D deficiency may lead to hypertension and anxiety.

#### 4.4.4. 5-Hydroxytryptamine (5-HT)

Gut dysbiosis suppresses 5-HT secretion and contributes to hypertension and anxiety. Tryptophan is produced by Lactobacillus and Bifidobacterium. In enterochromaffin cells, tryptophan hydroxylase 1 (TPH1) catalyzes the conversion of tryptophan to 5-hydroxytryptophan (5-HTP) in the colon. Tryptophan and 5-HTP can enter the brain and become precursors of 5-HT in the central nervous system [130]. Gut dysbiosis suppresses tryptophan secretion by decreasing the abundance of Lactobacillus and Bifidobacterium, suppressing the secretion of 5-HT [131]. The abatement of 5-HT exacerbates insulin resistance and leads to obesity and hypertension [132]. Consistently, a reduction in 5-HT levels accelerates the occurrence of anxiety. Additionally, selective serotonin reuptake inhibitors can increase the concentration of 5-HT in synapses, reduce anxiety, and control the risk of hypertension [133]. In summary, gut microbiotas intensify hypertension and anxiety by suppressing 5-HT secretion.

#### 4.4.5. Others

Gut dysbiosis increases intestinal MYC expression and aggravates hypertension and anxiety. MYC is a highly pleiotropic transcription factor that broadly affects cell proliferation, metabolism, angiogenesis, apoptosis, and differentiation [134]. Recent studies have suggested that gut dysbiosis increases MYC expression in the intestine. Subsequently, abundant intestinal MYC targets ceramide synthase 4 (Cers4) to stimulate ceramide synthesis and simultaneously decrease glucagon-like peptide 1 (GLP-1) secretion, facilitating obesity [135]. c-MYC, a member of the MYC family, induces anxiety by upregulating the expression of 5HT2AR and 5HT1AR [136].

These findings suggest that gut dysbiosis is a risk factor for hypertension and anxiety. Targeting gut microbiotas may be a novel therapeutic option for patients with comorbid hypertension and anxiety.

## 5. Conclusion

The increasing prevalence of comorbid hypertension and anxiety has a negative impact on treatment efficacy and quality of life. Understanding these population risk factors and physiological mechanisms will guide future clinical care for a population at increased risk for comorbid hypertension and anxiety. As discussed previously, the risk factors for comorbid hypertension and anxiety mainly include age, sex, smoking, alcohol abuse, obesity, lead exposure, and traffic noise. Aging is associated with an increased risk of comorbid hypertension and anxiety. Hormonal changes make women more likely to develop comorbid hypertension and anxiety. Moreover, undesirable lifestyle factors such as smoking, alcohol abuse, and obesity, play a crucial role in comorbid hypertension and anxiety. Additionally, people exposed to lead and traffic noises are highly prone to comorbid hypertension and anxiety. Hence, optimizing lifestyle or living environment remains the cornerstone in preventing and treating of hypertension and anxiety. Furthermore, IL-6, IL-17, ROS, and gut dysbiosis are potential mechanisms underlying comorbid hypertension and anxiety. An increase in IL-6, IL-17, and ROS levels can promote the occurrence of comorbid hypertension and anxiety. Changes in SCFs, TAMO, vitamin D, and 5-HT caused by gut dysbiosis play essential roles in the occurrence and development of hypertension complicated by anxiety. Similarly, the changes in SCFs, TAMO, vitamin D, and 5-HT caused by gut dysbiosis play important roles in the occurrence and development of hypertension complicated by anxiety. Therefore, screening for hypertension complicated by anxiety and assessing its risk factors is necessary. Further studies should focus on the mechanisms involved in comorbid hypertension and anxiety and identify drug targets that can treat hypertension complicated by anxiety, to improve the effectiveness of treatment.

## 6. Prospection

Autophagy is an evolutionarily conserved self-digestion process, essential for cellular homeostasis [137]. Autophagy declines with age and may increase the risk of age-related cardiovascular diseases [138]. Knockdown of autophagy-related genes (ATG) 5/7 suppresses Weibel–Palade bodies (WPB) in EC, thereby inhibiting endothelial diastolic function [139]. Similarly, mice suppressed essential gene (Ulk2) initiate autophagy and anxiety-like behavioral abnormalities [140]. Dysfunctional autophagy has been linked to gut dysbiosis and increased epithelial permeability [141]. Moreover, the deficiency of the mitochondrial autophagy-related gene (ATG16L1) increases the number of mitochondria and produces numerous ROS [142]. Given its role in endothelial dysfunction and ROS activation, autophagy may be a potential target for comorbid hypertension and anxiety. Owing to the lack of direct evidence, more studies are needed to explore the exact effects of autophagy in comorbid hypertension and anxiety.

## 7. Clinical Implications

The comorbidities of chronic mental and physical conditions complicate medical treatment and may expedite disease severity and progression. Anxiety and hypertension are common and often comorbid conditions treated during diagnosis and treatment process [143]. Therefore, healthcare systems and clinical care teams should focus on interventions to improve the screening, diagnosis, and timely treatment of hypertension comorbid anxiety. Effective, sustainable interventions are urgently needed to improve the screening and treatment of patients with comorbid hypertension and anxiety. This study summarizes the risk factors for hypertension and comorbid anxiety and provides a theoretical basis for medical staff to screen patients with comorbid hypertension and anxiety. Additionally, this study described some common underlying mechanisms of hypertension and anxiety, including IL-6, IL-7, ROS, and gut dysbiosis, which are expected to become therapeutic targets for patients with comorbid hypertension and anxiety.

## Figures and Tables

**Table 1:** The common risk factors for comorbid hypertension and anxiety.

Risk factors	The effects on hypertension	The effects on anxiety	Reference
Age	Endothelial dysfunction	Reduced 5-HT specific receptors	[19–22]

Sex	Drop in estrogen	Drop in estrogen	[23–25]

Smoking	Increased sympathetic activity	Nicotine-based withdrawal symptoms, perceived and objective health impairment	[26–29]
	Oxidative stress		

Alcohol abuse	Increased sympathetic activity	Abnormal brain function	[30–32]
	Activation of the renin-angiotensin-aldosterone system		

Obesity	Abnormal kidney function;	Senescent cells	[33, 34]
	Increase in tubular sodium reabsorption		

Lead	Increased sympathetic activity	Neuroinflammation	[35, 36]

Traffic noise	Intensive oxidative stress	Excessive stress hormone release	[37, 38]
